# Supplementary material for: Electric activity at magnetic moment fragmentation in spin ice
Source: Nat Commun. 2021 May 24;12:3047. doi: 10.1038/s41467-021-23380-w (PMC8144587; doi:10.1038/s41467-021-23380-w)
Supplement: Supplementary file 1 — Supplementary Information [file 41467_2021_23380_MOESM1_ESM.pdf]

SUPPLEMENTARY INFORMATION FOR THE PAPER "ELECTRIC ACTIVITY AT MAGNETIC MOMENT FRAGMENTATION IN SPIN ICE"
BY D. I. KHOMSKII

1. Creation and motion of defects in systems with moment fragmentation and their dipole character.

Point defects and the corresponding excitations in MF states can be created by some perturbations, e.g. by external defects etc., but they can also be thermally excited. In the later case they are created in pairs by reversing some spins. As discussed in the main text if one reverses the "special" spin say in Figs. 2(a) and 2(c), one creates a pair of (all-in) and (all-out) sites (triangles in kagome, tetrahedra in pyrochlore systems). The charges of these objects are, respectively, $\pm 3Q$ and $\pm 4Q$. Note right away that by making this pair of defects we simultaneously "destroy" two electric dipoles which originally formed a $(\boldsymbol{d}, -\boldsymbol{d})$ dimer pair on these sites. Another possibility is to reverse a "normal", not a special spin. In kagome systems this will interchange the monopole and antimonopole, i.e. create a $(\bar{\mu}, \mu)$ pair at the wrong places, in wrong sublattices. In pyrochlore systems such reversal of the usual spin will create two tetrahedra of the (2-in)–(2-out) type. As discussed in the main text, in both cases the defects created by that have some unpaired dipoles, though for example in pyrochlores the (2-in)–(2-out) sites themselves have no dipoles, see Fig. 3(c), (d).

By making such spin reversal we either create "supermonopoles" ((3-in) states in kagome, (4-in) states in pyrochlores), with the increased charge, or we instead decrease the charges at sites, e.g. by making non-monopole (2-in)–(2-out) tetrahedra in pyrochlores. One can speak in this case not of the charge of the defect itself, but of the *excess charge* of an excitation: one gets e.g. the "double monopole" of (4-in) type with charge $4Q$ instead of the original state of a monopole with charge $2Q$, i.e. the excess charge of such excitation is $+2Q$. This language is very convenient when considering the motion of such excitations.

Once created, these defects can start to move in a crystal by consecutively reversing some spins one after the other. But, in contrast to a similar motion of monopoles in regular spin ice [5], here these excitations move on the background of ordered monopoles. This makes their motion more complicated and more interesting.

If the motion of such defects would occur via simultaneous flipping of two spins [31], the defects can remain in their own sublattice. But the usual motion occurs via flipping of

one spin at a time. As shown in Fig. S1, in this case at each step the excitations moves from one sublattice to the other and by that always change their character (including their dipole structure). But they carry their excess charge with them. Thus, the excitation having the form of a tri-pole of Fig. S1(b) with charge $3Q$ and excess charge $+2Q$ moves to the neighbouring site, Fig. S1(c), and at this site its charge is $-Q + (+2Q) = +Q$, i.e. instead of the original antimonopole at this site (the blue triangle in Fig. S1(a), (b)) it becomes a monopole with charge $+Q$ (the magenta triangle in Fig. S1(c)). Simultaneously, as is seen form this figure, one creates the defect with three unpaired dipoles, as in Fig. 3(c) of the main text.

After that the defect has two options. Either it can move forward by reversing again the "special" spin, Fig. S1(d), or by reversing the remaining usual spin. In the first case one creates the tri-pole at a site of a monopole sublattice, i.e. one effectively moves the tri-pole from one site of this sublattice, red triangle in Fig. S1(b), to another site of the same sublattice, Fig. S1(d). This motion occurs via the intermediate "virtual" state when this excitation is on the "wrong" sublattice, Fig. S1(c), but in effect one can move in this way the excitation in its own sublattice. And one sees that by such motion we change the spins, and also electric dipoles, on the trajectory of the defect, but, similar to the case of the usual spin ice, all monopole states on such trajectory return to their original state, i.e. such string has no tension and there is no confinement. However if on the second step one would reverse not the special spin, as is done in going from (c) to (d), but the remaining usual spin of the magenta triangle in Fig. S1(c), moving the defect "to the right" in Fig. S1(c), then one would leave a trail of wrong monopoles on such trajectory. Thus, in contrast to monopoles in the usual spin ice, which can move without confinement in all directions, here the motion is partially restricted: at every second step the defect can only move in one direction, in the direction of the respective special spin. In effect not all trajectories of excitations are allowed, see Fig. S1. And this guarantees two things simultaneously: the absence of confinement, and also the absence of a trail of unpaired dipoles: all electric dipoles on the trajectory of the defect would be also paired in $(\boldsymbol{d}, -\boldsymbol{d})$ pairs — except maybe the ends of these strings, which would have unpaired dipoles if they are of the type of monopole on a wrong place in kagome and (2-in)–(2-out) defect in pyrochlores; these would have unpaired dipoles and would consequently contribute to the electric activity of the system.

Thus, in effect, when such point defects move on the MF background, they change the

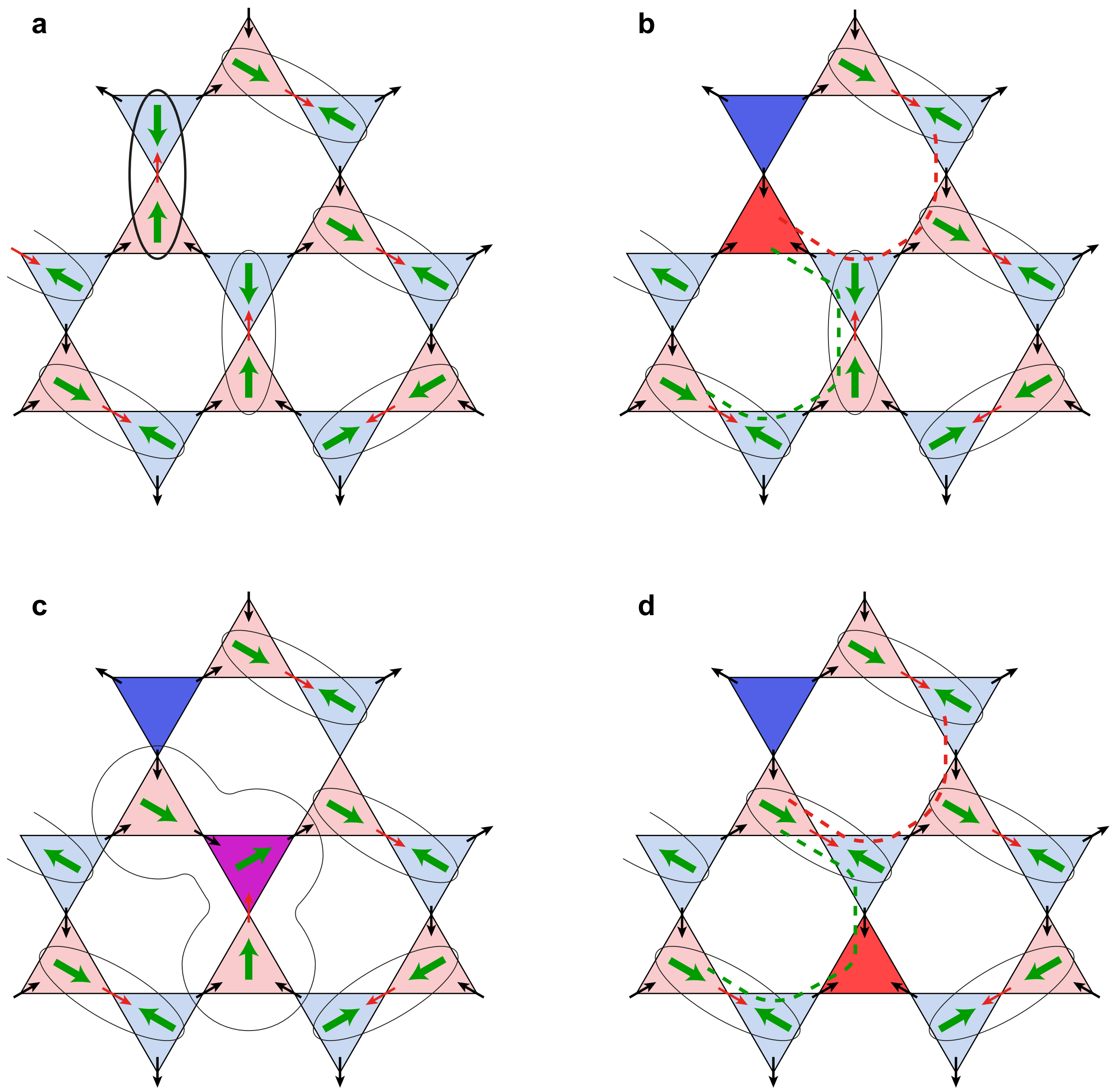

FIG. S1. Creation and motion of excitations in kagome systems with moment fragmentation and the corresponding distribution of electric dipoles. (a) Initial state with ordered monopoles, with dipoles (thick green arrows) paired in $(\boldsymbol{d}, -\boldsymbol{d})$ dimers (ovals). (b) Creation of triple monopoles and antimonopoles (red and blue triangles) by reversing one "special" spin (red arrow), with "annihilation" of two dipoles at the respective triangles. (c), (d) Motion of an excitation with the excess charge $+2Q$ by consecutive reversal of one spin at each step. In (c) it leads to the creation of a monopole (magenta triangle) in an antimonopole sublattice, with the creation of three unpaired dipoles, cf. Fig. 3(c). In (d) another "special" spin is reversed, and in effect the tri-pole (red triangle) shifts in its own sublattice leaving a trail of reversed spins (and paired dipoles) without confinement and without unpaired dipoles. By the green dashed line we show an allowed trajectory of the excitation, a string without confinement; by the red dashed line we show a forbidden trajectory, on which wrong sites (triangles) would appear.

character all the time, in kagome changing sublattices, and in pyrochlores from the double monopole to (2-in)–(2-out) states. I.e. this is actually the motion of the excitation with the charge $+2Q$ (or $-2Q$) on the periodic potential: such defects have different energies on different sublattices. Which state has lower and which has higher energy may depend on the particular situation. Thus one may think that in pyrochlores the (2-in)–(2-out) state would have lower energy than the (4-in) state. But the situation may be different for example in a MF state which exists in the kagome ice state in pyrochlores in [111] magnetic field slightly below phase transition to the fully-ordered state [24], see Fig. S2: the (3-in) (tri-pole) state of a kagome triangle (which is simultaneously a monopole (3-in)–(1-out) or (1-in)–(3-out) state of a tetrahedron) would have lower energy than the state of kagome monopoles in place of antimonopoles. In the first case all spins in kagome planes (their z-components) would point along the field, whereas for the second case two out of three such spins would be opposite to it. Thus in this case one should expect that the energy of an excitation would have lower energy when it is a "tri-pole". (Note, by the way, that such defects in kagome ice in a [111] field in pyrochlores would also have electric dipoles, shown in Fig. S2, whereas such kagome ice state itself has here no dipoles (every tetrahedron in this case has the (two-in)–(two-out) configuration without dipoles).) All in all, this is a very interesting situation deserving special attention (see also [31]), which, however, lies beyond the scope of the present paper.

2. Defects in pyrochlores with moment fragmentation.

Similarly to the case of kagome systems discussed in the main text, one can see that in pyrochlores the defects have in principle very similar properties, as to their dipole activity. In Fig. S3(a) we show the structure of the first type of point defects: the "supermonopole" state of the type (4-in) or (4-out). One sees from this figure that by recommuting the remaining spins one can form such defect without any free unpaired dipoles. But this is not the case for the other types of defect. One of them, the (2-in)–(2-out) state, shown in Fig. 3(d) of the main text, has, as is shown there, two unpaired dipoles attached to it. These two defects, (4-in) and (2-in)–(2-out) states, are the most important point defects, which, when they move, interchange all the time, see the previous section SI 1.

For other types of defects or textures, e.g. for domain walls, very important is the situation with two neighbouring tetrahedra both of monopole or antimonopole type. As one sees from Fig. S3(b), in this case a common spin is the in-spin for one tetrahedron, but it is the

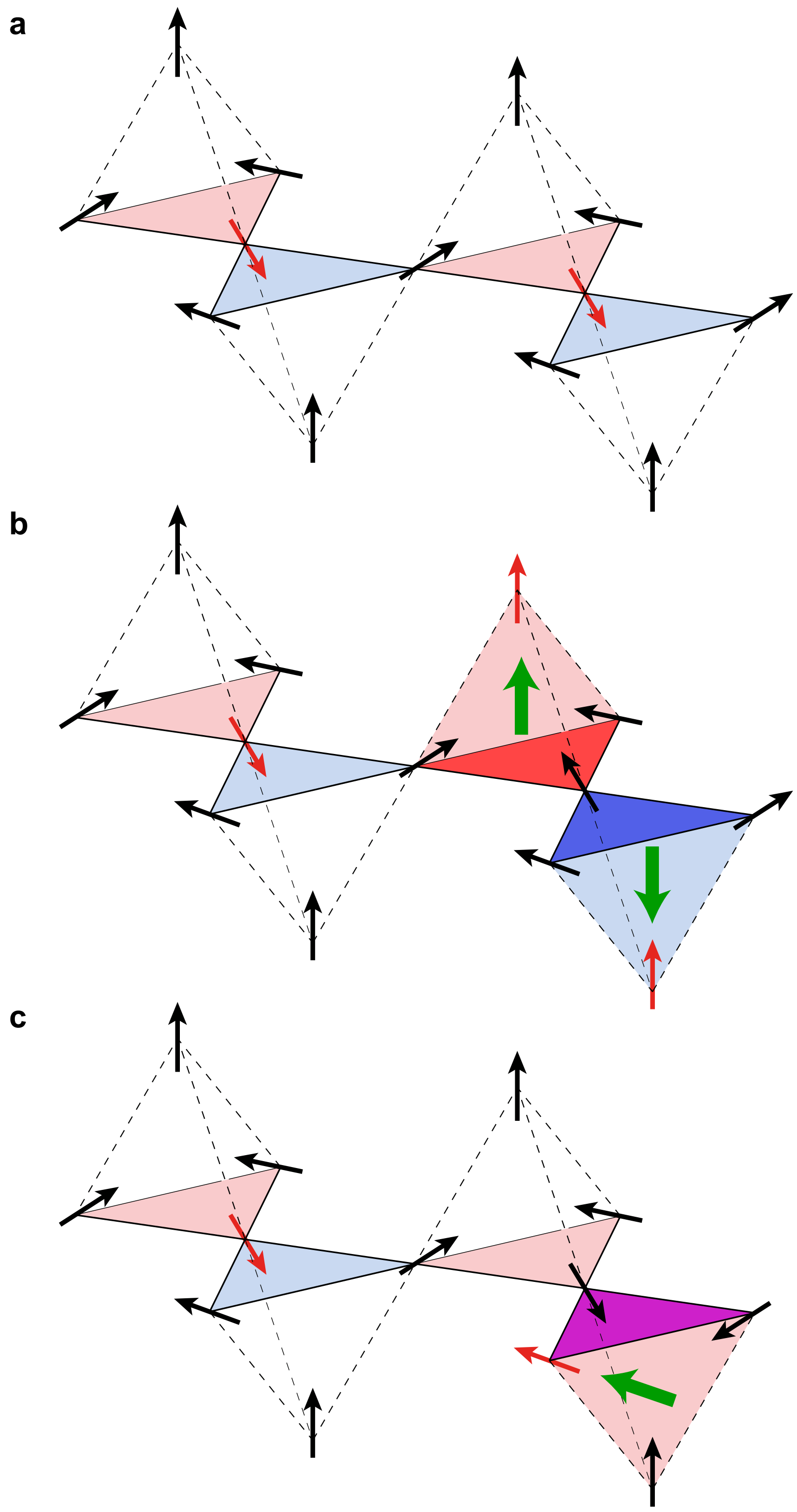

FIG. S2. Ground state and defects in kagome ice, obtained in pyrochlore systems such as $Dy_2Ti_2O_7$ in magnetic field $\boldsymbol{H} \parallel [111]$ slightly below the transition to a fully-ordered state. (a) Picture demonstrating that such a state is actually a MF state with ordered monopoles in the kagome plane. (b), (c) Two types of defects in the kagome plane: (b) tri-poles, (c) monopole in the wrong sublattice (magenta triangle). The electric dipoles at these defects are shown by thick green arrows.

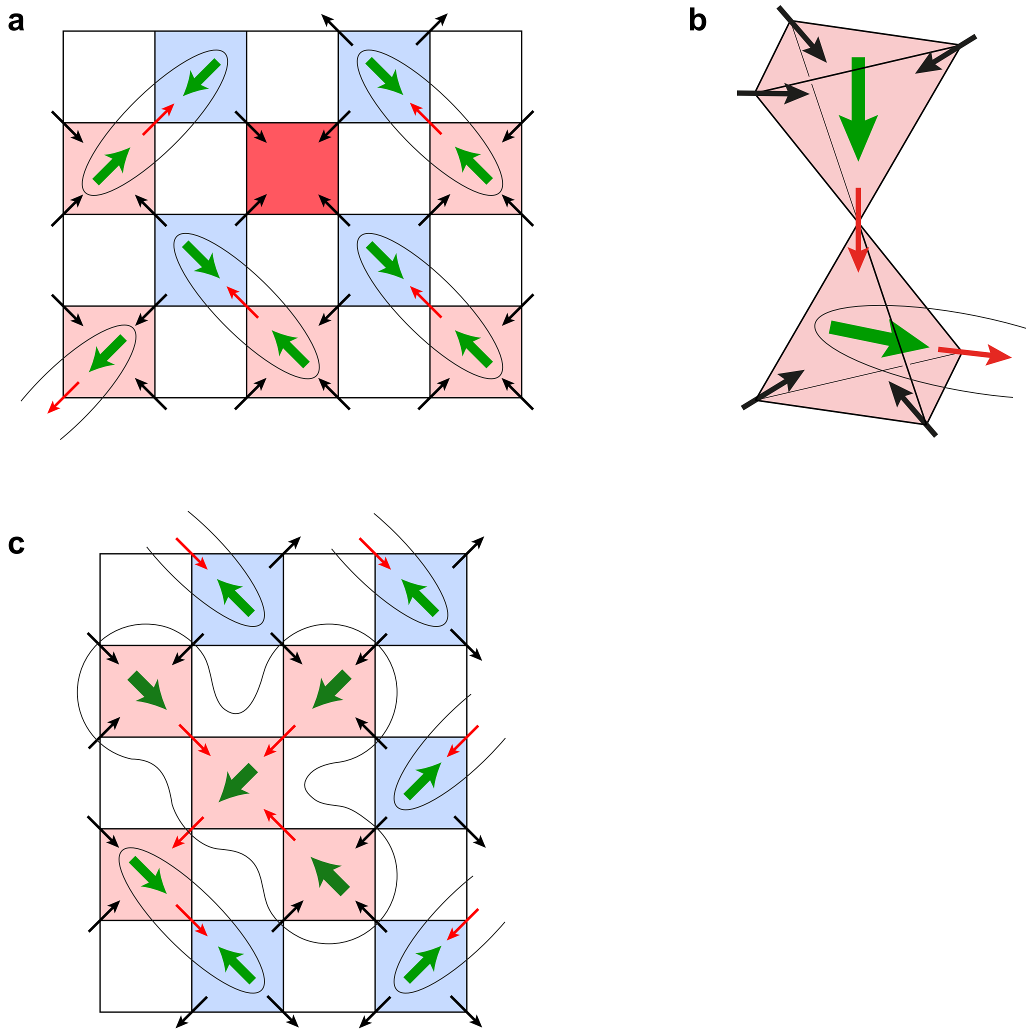

FIG. S3. Dipoles and defects in pyrochlore spin ice. (a) “Supermonopole” (4-in state). (b) Typical situation with two monopole neighbours. The common spin is the “special” out-spin for one monopole, and the dipole pointing in this direction is unpaired. (c) Point defect — the monopole in place of an antimonopole. Four unpaired dipoles would form at such defect.

out-spin (i.e. our “special” spin) for the other tetrahedron. In this second tetrahedron the electric dipole would point to this special spin, and it would not form a $(\boldsymbol{d}, -\boldsymbol{d})$ pair — it would definitely be an unpaired dipole.

Using this picture it is easy to understand that e.g. the isolated point defect — monopole in place of an antimonopole, would lead to the formation of even four such unpaired dipoles, Fig. S3(c): this “wrong” monopole tetrahedron would have four other neighbouring tetrahedra also with monopoles, and, by the arguments presented above, see Fig. S3(c), four out of these 5 tetrahedra would have unpaired spins. Such defect, which would have an excess

charge $\pm 4Q$, is of high energy, it requires the flipping of two spins, thus as an isolated defect it probably does not play an important role (but it can be created e.g. by the "fusion" of two usual excitations with the excess charge $2Q$). But such states play an important role in domain walls.

The same arguments show that there would appear unpaired dipoles at domain walls in pyrochlores with moment fragmentation. There would be in this case three types of such domain walls due to interchange of μ and $\bar{\mu}$ sublattices in half of the sample, Fig. S4. Domain walls of the first type, similar to domain walls of type 1 in kagome systems, are the walls going through triangular sites in (111) layers in pyrochlore lattice, Fig. S4(a). At such walls the neighbouring tetrahedra would have one common site, and dipoles would point perpendicular to such domain wall, i.e. in the [111] direction.

Another domain wall is also perpendicular to the [111] axis, but runs through the kagome (111) plane, Fig. S4(b). In this case, similar to the domain wall of type 2 in kagome systems, we would also have neighbouring monopole tetrahedra, which again would have unpaired dipoles, but these dipoles would point more or less along the domain wall plane (tilted from it). And finally, the third type of domain walls in pyrochlores with MF, Fig. S4(c), are those perpendicular to the cubic [001] axis (or other equivalent cubic axes). One easily sees that unpaired dipoles in this case would also point more or less along the plane of a domain wall, as in the previous case. Thus, in general, the properties of defects in pyrochlores with MF are very similar to those in kagome systems, considered in the main text, and most of the defects would have an electric activity and could in principle be controlled by an electric field.

3. Currents and orbital moments in a moment fragmentation state.

As is clear from Eqs. (2), (3), nontrivial effects such as spontaneous orbital currents and corresponding orbital moments exist only for magnetic structures with noncoplanar spins. Consequently they are absent e.g. in kagome systems with spins in the xy-plane. There exists however a very interesting group of kagome systems — "kagome-from-pyrochlores" (or "tripod kagome") obtained by nonmagnetic dilution of spin-ice pyrochlores, e.g. $Dy_3SbMg_2Sb_2O_{14}$ [29]. One can visualise these systems as ordered [111] kagome layers of magnetic ions, triangular [111] layers being occupied by nonmagnetic Sb, see Fig. S5.

That is, in the original magnetic tetrahedra with Ising ions (spins pointing in or out of tetrahedra) one makes tetrahedra in which the apical magnetic ions are replaced by

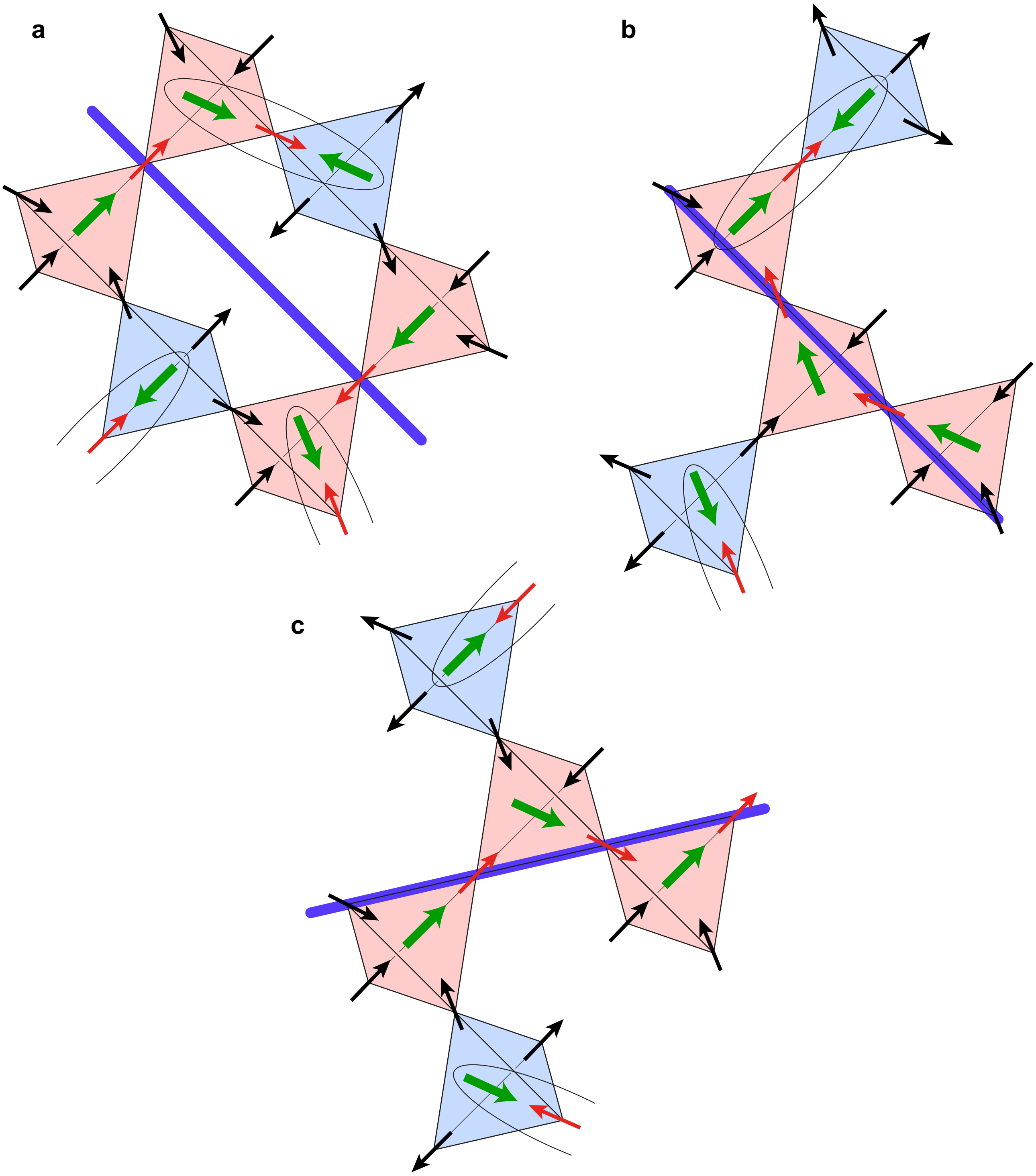

FIG. S4. Domain walls in pyrochlores with moment fragmentation, with corresponding electric dipoles. (a) Domain wall running through triangular (111) plane. (b) Domain wall running through kagome (111) plane. (c) Domain wall at (001) plane.

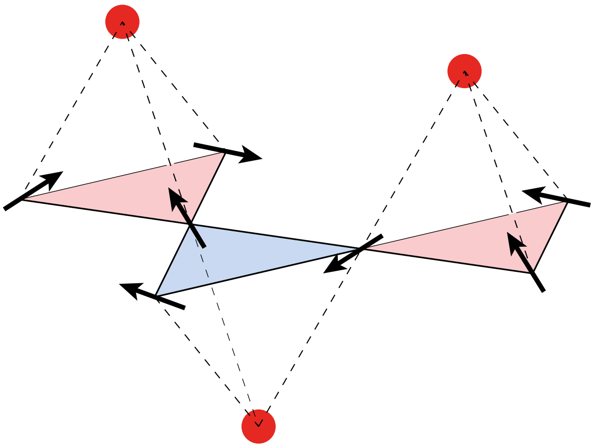

FIG. S5. Structure of a “kagome-from-pyrochlore” system, e.g. $Dy_3SbMg_2Sb_2O_{14}$ [29]. Red balls are nonmagnetic ions.

nonmagnetic ones, but the spin direction of the remaining magnetic ions is the same as before: spins point either in or out of M_3M' (here Dy_3Sb) tetrahedra, see Fig. S5.

According to Eqs. (2), (3) the scalar spin chiralities and corresponding currents and orbital moments are of one sign (e.g. currents clockwise, orbital moments up) for monopoles, and are opposite for antimonopoles. In regular kagome spin ice (“kagome-from-pyrochlores”) they are random, but in the moment fragmentation state with full $(\mu, \bar{\mu})$ ordering these currents and orbital moments are also fully ordered, Fig. S6. In that sense currents and orbital moments in MF kagome systems are different from electric dipoles in those: dipoles form pairs, but are random and dynamic, but currents and orbital moments are fully ordered together with monopoles themselves.

The situation with spontaneous currents and orbital moments in pyrochlores, with and without MF, is a bit more tricky than that in kagome systems, but conceptually similar. One can show that for spin ice tetrahedra there would exist nonzero currents even in a pure spin-ice case (2-in)–(2-out) without monopoles. Indeed, using the expressions (2), (3), one can show that the currents in this case would have a trajectory shown in Fig. S7(a), i.e. there would exist an orbital moment pointing from the edge with 2-in to the edge with 2-out spins. In effect, similar to the kagome case, the orbital moment would point along (here antiparallel) the net spin moment of such tetrahedra.

The same rule actually applies to tetrahedra with monopoles or antimonopoles. The currents in this case would only flow along a triangle with (3-in) or (3-out) spins, Fig. S7(b)

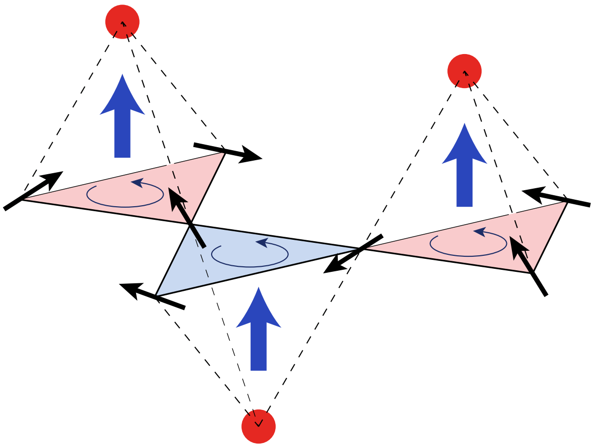

FIG. S6. Orbital currents and orbital magnetic moments in "kagome-from-pyrochlore" spin ice in a moment fragmentation state. Currents (thin rings in triangles), clockwise in monopoles, anticlockwise in antimonopoles (looking from outside of tetrahedra!), and orbital moments (thick blue arrows) in triangles. In contrast to electric dipoles, here the spontaneous currents and orbital moments are fully ordered in a MF state.

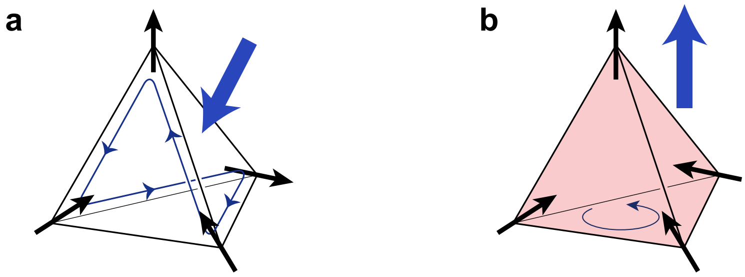

FIG. S7. Orbital currents and orbital magnetic moments in pyrochlore spin ice. (a) The usual (2-in)—(2-out) tetrahedra. (b) Currents and orbital moments at monopoles and antimonopoles (for the negative coefficient c in Eq. (2)). Note that in both cases orbital magnetic moments are parallel (or antiparallel) to the net spin moment of corresponding tetrahedra.

(the currents on the other three edges of such tetrahedra, each belonging to two triangles, would cancel). And the corresponding orbital moment would point again in the direction of the total spin of corresponding tetrahedra.

The situation with currents and orbital moments in pyrochlores with moment fragmentation is different from that in a kagome ice. When we look at a pair of neighbouring tetrahedra with opposite monopole charges, Fig. S8, and with the "special" spin at the common site between those, we indeed see that the electric dipoles of these tetrahedra are

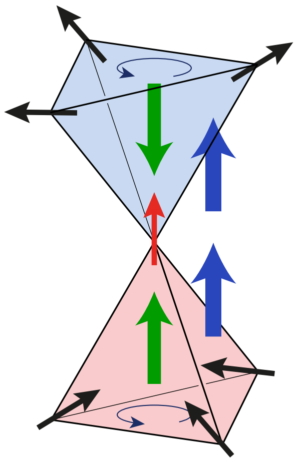

FIG. S8. Currents and orbital magnetic moments for a typical configuration in pyrochlore spin ice with moment fragmentation. Green arrows are dipole moments, thick blue arrows are orbital moments.

opposite and form a $(\boldsymbol{d}, -\boldsymbol{d})$ pair oriented along the corresponding axis (one of [111] axes of pyrochlore lattice). But the currents here, according to Eq. (3), would run along triangles in the basal plane, opposite to the "special" spin, but they would run (looking from outside!) in the opposite direction, clockwise and counterclockwise. Consequently the orbital moments created by these currents would also be parallel and would point in the same direction as the total spin of respective tetrahedra. Thus for monopoles the orbital moments would be parallel to electric dipoles, $\boldsymbol{L}_i \parallel \boldsymbol{d}_i$, but for antimonopoles they would point in opposite directions, Fig. S8. When spins themselves change (keeping the MF state), both $(\boldsymbol{d}, -\boldsymbol{d})$ and $(\boldsymbol{L}, \boldsymbol{L})$ pairs move and would change orientation. This is in contrast to the case of kagome systems (kagome-from-pyrochlore), in which in the fragmentation state currents and orbital moments are long-range ordered together with monopoles themselves, whereas the $(\boldsymbol{d}, -\boldsymbol{d})$ pairs fluctuate together with spins.
